# Complete mitochondrial genome of the snow petrel, *Pagodroma nivea*

**DOI:** 10.1080/23802359.2020.1820389

**Published:** 2020-09-15

**Authors:** Jong-U Kim, Jeong-Hoon Kim

**Affiliations:** Korea Polar Research Institute, Incheon, South Korea

**Keywords:** Mitogenome, *Pagodroma nivea*, sea bird, snow petrel

## Abstract

The snow petrel, *Pagodroma nivea* is a small sea bird endemic to Antarctica and the Southern Ocean. Herein, we assembled a complete mitochondrial genome of the snow petrel as a first revealed genetic resource in Pagodroma family. The mitogenome is 17,279 bp in length and consists of 13 protein-coding genes (PCGs), 22 *tRNA* genes, and two *rRNA* genes. Base composition is 31.3% A, 25.3% T, 30.1% C, and 13.2% G with CG content of 43.4%. These results will provide a useful basis for further genetic and phylogenetic studies of this species.

The snow petrel (*Pagodroma nivea* Forster, G, 1777) is a small sea bird endemic to Antarctica and the Southern Ocean (Croxall et al. 1995). The species is a member of the family Procellariidae, order Procellariiformes, and is the only species representing the genus *Pagodroma*. It is divided into two subspecies (*P. nivea* and *P. major*) but taxonomic status remains controversial (Carrea et al. 2019). Snow petrels exhibit broad geographic distribution and a large, relatively stable global population (Birdlife International 2020). Despite this, few genetic studies have been conducted (Croxall et al. 2012). Also, mitochondrial genome was not reported in Pagodroma family until now. Thus, elucidation of the snow petrel mitochondrial genome will provide a basis for improved understanding of their phylogeny and evolution.

In this study, we sequenced and analyzed the complete mitogenome of *P. nivea* (GenBank: MT726204). A single specimen (proof number SP1) was collected in Bransfield Strait, Antarctica (62°25′52.8′′S, 58°42′20.2′′W), on 12 April 2018 and stored at the Korea Polar Research Institute (KOPRI), Incheon, South Korea. Total genomic DNA was extracted from muscle tissue using a DNeasy Blood and Tissue kit (Qiagen, Hilden, Germany). An Illumina paired-end (PE) library was prepared according to the manufacturer’s instructions, and sequencing was performed using an Illumina sequencing platform by commercial company (Phyzen, Seongnam, South Korea). High-quality PE reads of about 1.03 Gb were obtained after trimming and were *de novo* assembled using CLC genome assembler version 4.21 (CLC Inc., Aarhus, Denmark). From the initially assembled contigs, those with mitogenome-derived sequences were further processed to generate a single draft sequence, as previously described (Lee et al. 2018). The draft sequence was manually corrected and gap-filled *via* a series of PE read mappings. The final complete mitogenome sequence was annotated using GeSeq (https://chlorobox.mpimp-golm.mpg.de/geseq-app.html) and manually curated using the Artemis annotation tool (Rutherford et al. 2000) with NCBI BLASTN searches (https://blast.ncbi.nlm.nih.gov). A maximum likelihood (ML) phylogenetic tree was constructed: multiple alignment of 13 protein-coding gene (PCG) sequences in mitochondrial genomes were performed using MAFFT (Katoh and Standley 2013) and used Tamura-Nei model and 1000 bootstrapping options via MEGA version 7.0 program (Pennsylvania State University, USA) (Kumar et al. 2016).

The resulting complete *P. nivea* mitochondrial genome sequence is 17,279 bp in length, including 13 PCGs, 22 *tRNA* genes, and two *rRNA* genes. Nucleotide composition is as follows: 31.3% A, 25.3% T, 30.1% C, and 13.2% G with a 43.4% GC content. Phylogenetic relationship revealed that *P. nivea* and *Daption capense* are a sister species and *Pterodroma brevirostris* and *Oceanodroma castro* belong to the same lineage (Figure 1).

**Figure 1.**
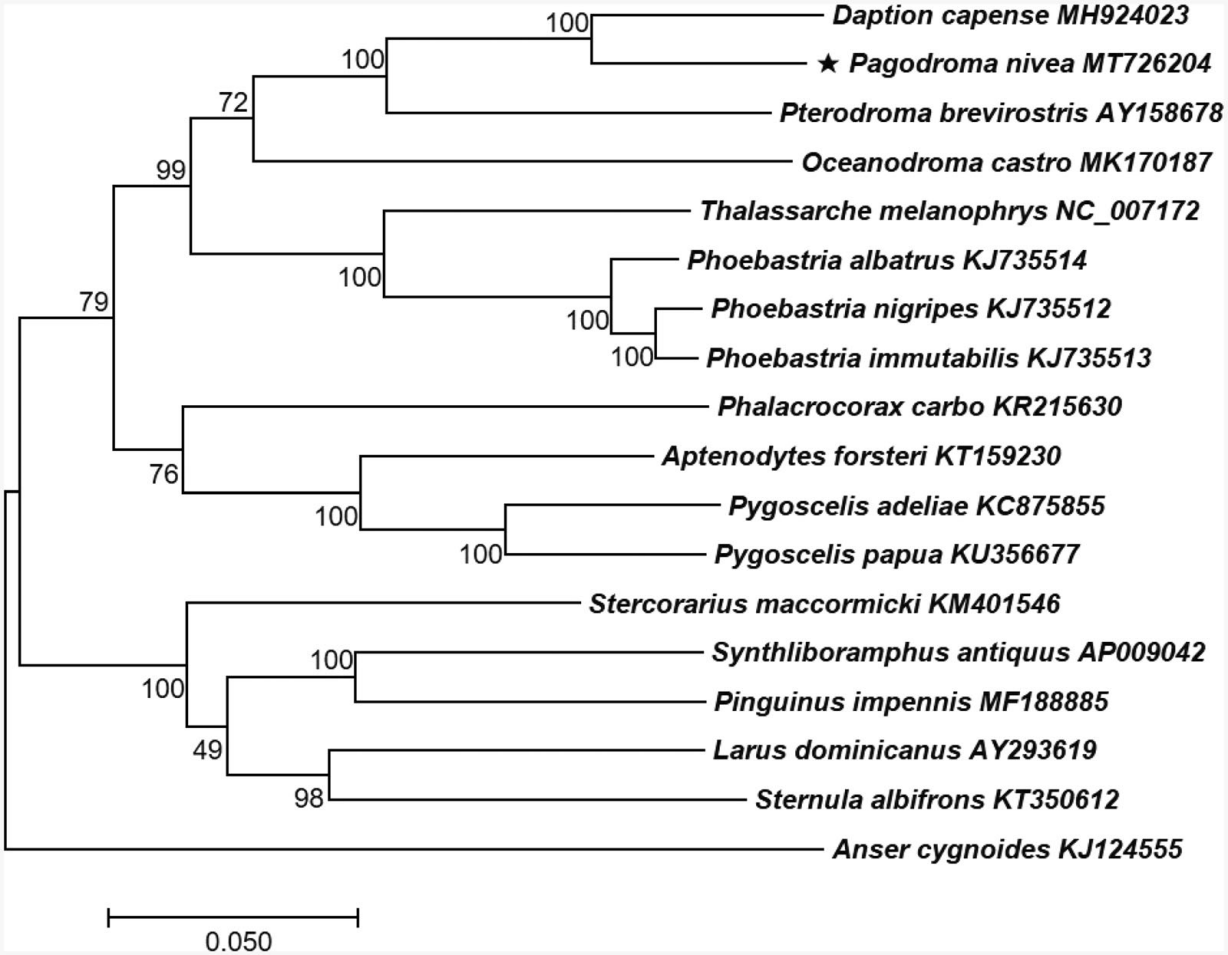
Phylogenetic tree of *P. nivea* and 17 related taxa. A total of 13 PCGs in mitochondrial genome were aligned and used to generate a maximum likelihood phylogenetic tree. The numbers in the nodes indicate bootstrap support values (>50%) from 1000 replicates .

## Data Availability

The data that support the finding of this study are openly available in NCBI at (https://www.ncbi.nlm.nih.gov), reference number [MT726204] and available from the corresponding author.

## References

[CIT0001] BirdLife International. 2020. Species factsheet: *Pagodroma nivea*. [accessed 2020 Jul 03]. http://www.birdlife.org.

[CIT0002] Carrea C, Burridge CP, Wienecke B, Emmerson LM, White D, Miller KJ. 2019. High vagility facilitates population persistence and expansion prior to the Last Glacial Maximum in an Antarctic top predator: the snow petrel (*Pagodroma nivea*). J Biogeogr. 46(2):442–453.

[CIT0003] Croxall JP, Butchart SHM, Lascelles B, Stattersfield AJ, Sullivan B, Symes A, Taylor P. 2012. Seabird conservation status, threats and priority actions: a global assessment. Bird Conserv Int. 22(1):1–34.

[CIT0004] Croxall JP, Steele WK, Mcinnes SJ, Prince PA. 1995. Breeding distribution of the snow petrel *Pagodroma nivea*. Marine Ornithol. 23:69–100.

[CIT0005] Katoh K, Standley DM. 2013. MAFFT multiple sequence alignment software version 7: improvements in performance and usability. Mol Biol Evol. 30(4):772–780. 23329690 10.1093/molbev/mst010 PMC3603318

[CIT0006] Kumar S, Stecher G, Tamura K. 2016. MEGA7: molecular evolutionary genetics analysis version 7.0 for bigger datasets. Mol Biol Evol. 33(7):1870–1874. 27004904 10.1093/molbev/msw054 PMC8210823

[CIT0007] Lee HO, Choi JW, Baek JH, Oh JH, Lee SC, Kim CK. 2018. Assembly of the mitochondrial genome in the campanulaceae family using Illumina low-coverage sequencing. Genes. 9(8):383. 10.3390/genes9080383 PMC6116063 30061537

[CIT0008] Rutherford K, Parkhill J, Crook J, Horsnell T, Rice P, Rajandream MA, Barrell B. 2000. Artemis: sequence visualization and annotation. Bioinformatics. 16(10):944–945. 11120685 10.1093/bioinformatics/16.10.944

